# Case report and literature review: A young man with giant intra-abdominal Ewing sarcoma

**DOI:** 10.1097/MD.0000000000039983

**Published:** 2024-10-04

**Authors:** Guang Yang, Lining Huang, Jianwu Wu, Bo Huang, Cong Zhang, Song Li, Feng Wang, Xinwei Jiang

**Affiliations:** a Gusu School, Nanjing Medical University, Nanjing, China; b Department of Hepatobiliary Surgery, the Affiliated Suzhou Hospital of Nanjing Medical University, Suzhou, China.

**Keywords:** chemotherapy, extraosseous Ewing sarcoma, genetic analysis, immunohistochemistry, intestinal mesenchymal tumor, multivisceral resection

## Abstract

**Rationale:**

Extraosseous Ewing sarcoma (EES) is a rare manifestation within the Ewing sarcoma tumor family (ESFT). Its clinical manifestations lack specificity, intestinal obstruction is the main symptom but can also present with abdominal pain, gastrointestinal bleeding, and other discomforts, making it prone to misdiagnosis as intestinal mesenchymal tumor.

**Patient concerns:**

A 29-year-old male was admitted to the hospital with intestinal obstruction symptoms and abdominal CT suggesting “left abdominal occupation.”

**Diagnosis:**

The patient was initially misdiagnosed as intestinal mesenchymal tumor, and was later definitively diagnosed as abdominal Ewing sarcoma by postoperative pathology and genetic testing.

**Interventions:**

Due to the patient’s surgical indication, surgical resection with exploratory laparotomy was performed and then the patient underwent systemic chemotherapy.

**Outcomes:**

Intraoperatively, we found a 15-cm tumor originating from the proximal jejunum, with invasion into the peritoneum, duodenum, jejunum, and colon. Finally, the pathological report revealed Ewing sarcoma.

**Lessons:**

Giant abdominal Ewing sarcoma with a diameter of 15 cm is rare. Considering postoperative pathology and genetic testing, abdominal Ewing sarcoma was suspected. The patient was successfully treated using surgery.

## 1. Introduction

Extraosseous Ewing sarcoma (EES) is a virulent tumor, notorious for its high rates of local recurrence and distant metastasis. To our knowledge, only a limited number of intra-abdominal Ewing sarcomas have been documented, manifesting in diverse locations including the gastrointestinal tract, kidneys, adrenal glands, pancreas, supraperitoneum, and retroperitoneum. Up to date only 8 cases of extraosseus Ewing sarcoma arising in jejunum are reported in the literature (Table 1). In this case, abdominal distension was the first symptom, but there was no definitive evidence to support the diagnosis of Ewing sarcoma. An abdominal CT scan initially suggested an intestinal mesenchymal tumor combined with the patient’s gastrointestinal symptoms, surgical resection was performed. Interestingly, postoperative specimens enabled the identification of Ewing sarcoma through light microscopy, immunohistochemistry, and molecular genetic evaluations.

**Table 1:** Reports of Ewing sarcoma involving the jejunum.

Author, year	Age/sex	Described location	Treatment	Survival months
Saranganathan, 2001	13/M	Jejunum	Surgery	12+
Kim, 2007	63/M	Terminal ileum and jejunum	Surgery→Systemic therapy	N/A
Padma, 2015	22/F	Distal jejunum	Surgery	N/A
Kim, 2017	9/F	Jejunum	Systemic therapy→Surgery →Adjuvant therapy	N/A
Cantu, 2019	67/F	Jejunum	Surgery	3+
Yagnik, 2019	42/M	Jejunum	Surgery→Systemic therapy	+
Kolosov, 2020	30/F	Jejunum	Surgery	2
Shadhu, 2021	55/F	Jejunum	Surgery→Systemic therapy	+
Our case, 2023	29/M	Proximal jejunum	Surgery→Systemic therapy	6+

N/A = not available.

+ = no information of death upon the last follow up.

## 2. Case presentation

**Figure 1.**
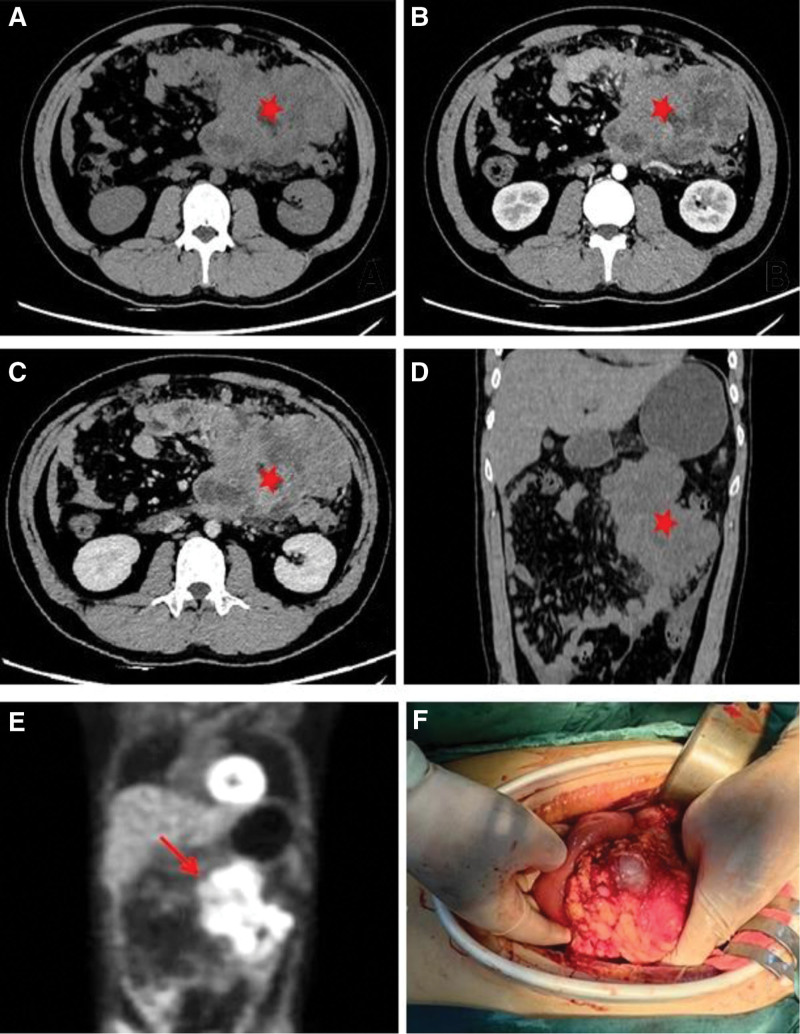
Images of abdominal computed tomography and positron emission tomography (PET), macroscopic specimen during operation. (A) Plain scan; (B) arterial phase; (C) venous phase; (D) coronary scan; (E) DWI diffusion image; (F) intraoperative view.

**Figure 2.**
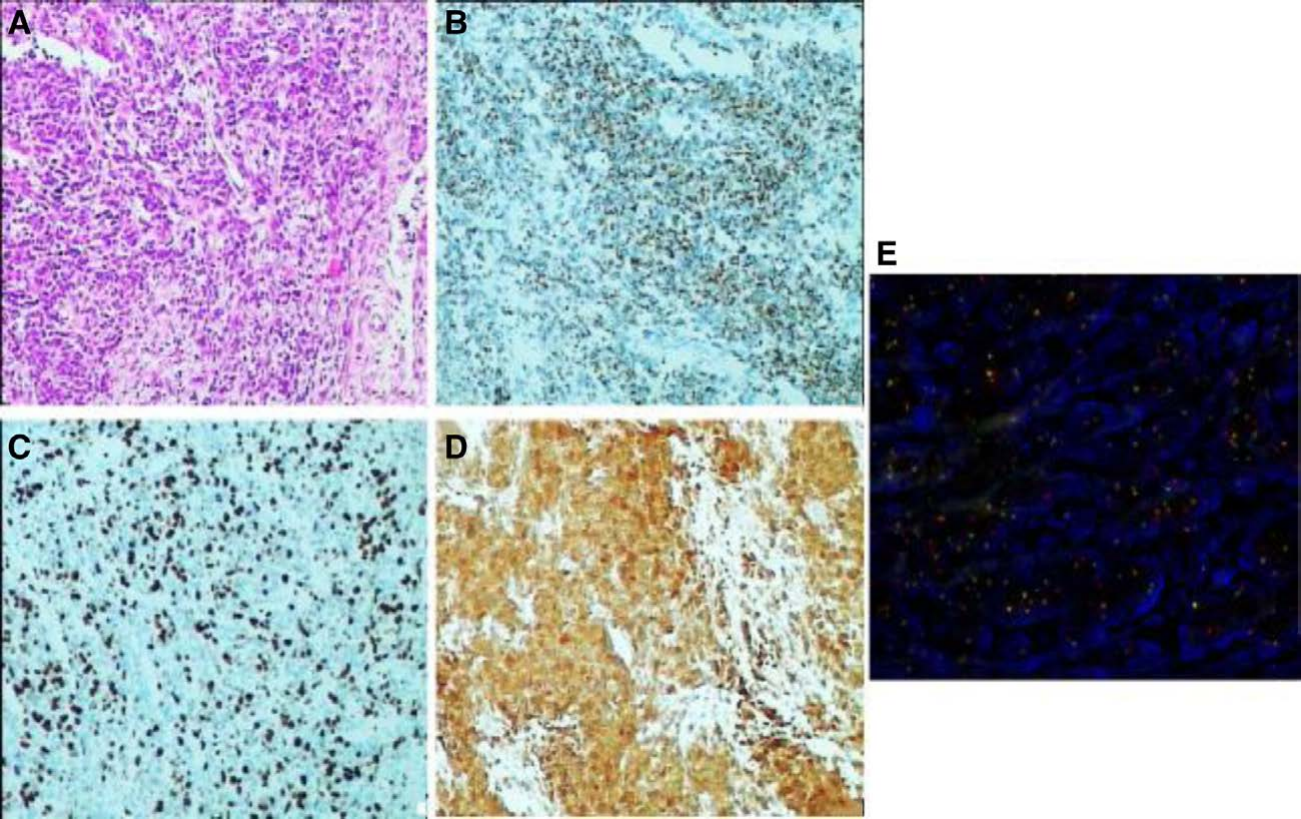
Pathological findings. (A–D) HE and immunohistochemical staining of postoperative tumor tissue: (A) HE staining; (B) CK18(+); (C) Ki-67(+); (D) NSE(+). (E) Fluorescent in situ hybridization (FISH) results show a balanced translocation involving chromosomes 11 and 22, fusing portions of the EWS gene on 22q12 (red signal) with the FLI-1 gene on 11q24 (green signal), thus creating a novel fusion gene with oncogenic properties.

**Figure 3.**
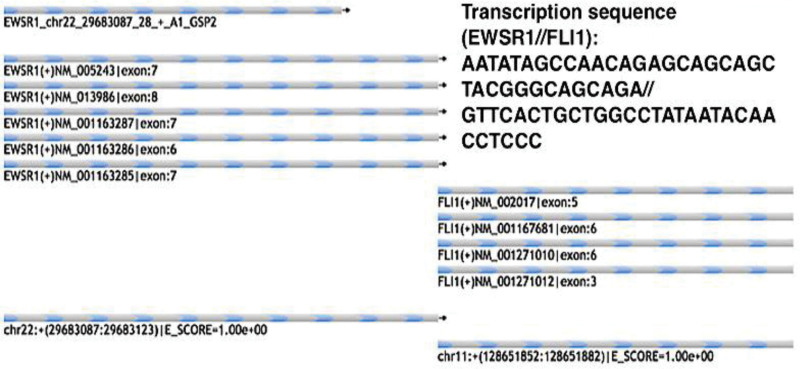
The in-frame fusion between genes EWSR1 Exons 1 to 7 (NM_ 005243) and FLI-1 Exons 5 to 9 (NM_002017).

**Figure 4.**
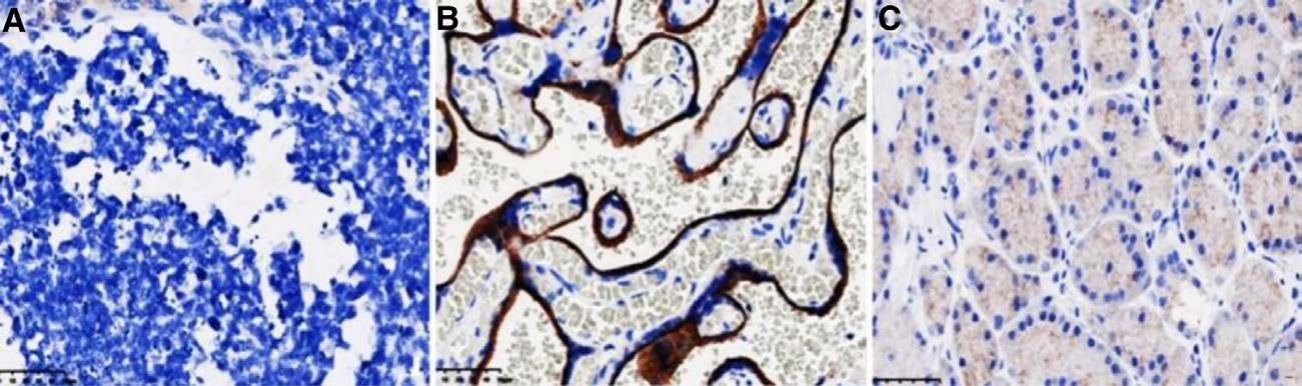
Immunohistochemistry of PD-L1 protein in postoperative specimen from Beijing Genomics Institution. (A) Microscopic image of postoperative specimen. (B) Positive control. (C) Negative control.

A 29-year-old male was hospitalized due to abdominal distension. Followed by severe vomiting and constipation intestinal obstruction symptoms, in addition to gastrointestinal bleeding and anemia. Comprehensive abdominal CT (both plain and enhanced) showed a 13.2 × 9.9 × 15 cm mass on the left side, suggestive of a possible mesenchymal origin, accompanied by multiple lymph node metastases within the abdominopelvic region, as well as gastric wall thickening in the gastric sinus area (Fig. 1A–D). To determine the presence of distant metastases. The 18F-FDG positron emission tomography (PET)-CT illustrated an anomalous giant mass in the left mid-upper abdomen, characterized by cystic degeneration, necrosis, and elevated FDG metabolism. Additional findings included thickening of the abdominopelvic tracts and omentum, presenting multiple nodules with irregular FDG metabolism. These features were indicative of malignant infiltration into adjacent organs, including the small intestine, the lateral wall of the gastric lesser curvature and multiple metastases in the abdominopelvic cavity. Notably, other organs did not exhibit abnormal FDG metabolism (Fig. 1E).We did not perform a puncture biopsy due to indications for surgical intervention such as obstruction and bleeding of the tumor, as well as the technical risks of ultrasound-guided puncture biopsy and the limited number of sites to be sampled. The patient’s overall condition was acceptable, according to the preoperative laboratory results (Table 2). No obvious abnormalities were found in tumor markers. Although the tumor was huge, invading the peritoneum and approaching organs, it did not invade important celiac arteries (celiac trunk artery, superior mesenteric artery, common hepatic artery), veins (superior mesenteric vein, portal vein) and distant organ from the CT image and PET-CT. Therefore, either R0 or R1 tumor resection or effective tumor reduction surgery can alleviate the clinical symptoms of patients and may improve their long-term prognosis. During the operation, the tumor was observed originating from the proximal jejunum (Fig. 1F), with invasion into the peritoneum, duodenum, jejunum, and colon. The abdominal mass and the affected tissues were surgically removed for histopathological assessment. The pathology revealed a small round cell malignant tumor. Immunohistochemical studies showed that CD99, Ki-67, EMA, CKpan, CK18, Vim, NSE, SDHB, BRG1, Syn were positive, CD56, CgA, CD117, DoG1, CD34, TTF-1, MyoD1, CK5/6, S-100, Myogenin, LCA, WT-1, Sall4, TLE-1, CR were negative (Fig. 2A–D). Fluorescent in situ hybridization, performed at the consulting institution, showed EWSR1 gene rearrangement in 90% of cells (Fig. 2E). Additionally, an Archer FusionPlex-targeted RNA sequencing analysis was performed at the same institution (Fig. 3). The Archer analysis software V5.0 used for data analysis identified the EWSR1-FLI-1 fusion transcript thus indicating a translocation t(11;22) (q24;q12). Together with the microscopic morphology and genetic findings, a diagnosis of Ewing sarcoma was ascertained. For the next step of treatment, the resected tissue underwent PD-L1 immunohistochemical analysis. The tumor cell positive score (TPS) was 0%, and the combined positive score (CPS) was < 1 (Fig. 4 and Table 3), indicating that the patient might not benefit from PD-1/PD-L1 inhibitor therapy. Postoperative treatment with alternating VDC (Vincristine, Doxorubicin, Cyclophosphamide) and i.e. (lfosfamide, Etoposide) was carried out, and the patient has undergone 7 courses of chemotherapy so far, and is now in stable condition.

**Table 2:** The preoperative laboratory data.

Hematology	WBC	7.52 × 10^9^/L
	RBC	3.23 × 10^12^/L↓
	Hb	77g/L↓
	PLT	555 × 10^9^/L↑
Biochemistry	TP	66.3 g/L
	AIb	34.54 g/L↓
	TBIL	9.2 μmol/L
	DBIL	2.2 μmol/L
	GPT	20 U/L
	GOT	12 U/L↓
	LDH	188 U/L
	ALP	109 U/L
	γ-GTP	82 U/L↑
	CHE	256 U/L
	BUN	4.44 mmol/L
	Cre	76.7 μmol/L
	Na	139.6 mmol/L
	K	4.23 mmol/L
	Cl	105 mmol/L
	Ca	2.19 mmol/L
	FPG	5.40 mmol/L
	TG	0.97 mmol/L
	TC	3.99 mmol/L
Coagulation	PT	13.1 s↑
	APTT	31.5 s
Serology	CRP	157 mg/L↑
Tumor marker	CEA	1.56 ng/mL
	AFP	3.37 ng/mL
	CA19-9	2.48 U/mL
	CYFRA21-1	0.95 ng/mL

AFP = Alpha-fetoprotein, Alb = albumin, ALP = alkaline phosphatase, APTT = activated partial thromboplastin time, BUN = blood urea nitrogen, CA19-9 = carbohydrate antigen 19-9, CEA = carcinoembryonic antigen, CHE = cholinesterase, Cre = creatinine, CRP = C-reactive protein, CYFRA21_1 = Cytokeratin 19 fragment, DBIL = direct bilirubin, FPG = fasting plasma glucose, GOT = glutamic oxaloacetic transaminase, GPT = glutamic pyruvic transaminase, Hb = hemoglobin, LDH = lactate dehydrogenase, PLT = platelet count, PT = prothrombin time, RBC = red blood cell, TBIL = total bilirubin, TC = total cholesterol, TG = triglyceride, TP = total protein, WBC = white blood cell, γ-GTP = gamma glutamyl transpeptidase.

**Table 3:** PD-L1 protein expression detection from Beijing genomics Institution.

	Value	Result
Tumor cell proportion score (TPS)	0%	Negative
Combine positive score (CPS)	<1	

## 3. Discussion and conclusions

The incidence of EES is 0.4 per 1 million, which is 10 times higher than that of Ewing sarcoma of the bone.^[1]^ Its incidence has a bimodal distribution, peaking in people under 5 years of age and over 35 years of age.^[2]^ Although EES can arise in any anatomical soft tissue, the predominant sites include the upper thigh, gluteal region, upper arm, and shoulder.^[3]^ Conversely, metastatic progression primarily targets the pulmonary system, skeletal framework, and bone marrow. Consequently, the clinical manifestations of EES are contingent on both its origin and metastatic locales, with 25% of cases revealing metastatic sites at initial diagnosis.^[4]^

With regard to the diagnosis of EES, since there are no specific markers for EES, a spectrum of immunohistochemical markers has been used to study it. These encompass the CD99 antigen – a type 1 glycoprotein revered for sensitivity but lacking specificity; neuromarkers such as S-100 protein and synaptophysin, and FLI-1,a DNA-binding transcription factor implicated in the t(11;22) translocation, offering heightened specificity over CD99.^[5]^ Characteristic chromosomal translocations of the ESFT include t(11;22)(q24;q12) and t(21;22)(q22;q12).^[6]^ The former is prevalent in 90% of cases, resulting in the fusion of the FLI gene at 11q24 with the EWSR1 gene at 22q12. This merger produces the EWS/FLI-1 transcript, incorporating the DNA-binding domain of FLI-1 in lieu of EWS’s RNA-binding domain.^[6]^ The latter translocation amalgamates EWSR1 with ERG, an alternative DNA-binding entity, crafting an oncogenic transcription factor impeding apoptotic mechanisms.^[7]^ Reports also shed light on rarer translocations, all converging on the EWSR1 gene located on chromosome 22.^[7]^ In our case, immunohistochemistry CD99(+),Syn(+),Vim(+), NSE(+), and CD117(−) were consistent with EES rather than intestinal mesenchymal tumor and the diagnosis of EES was also confirmed by positive results of genetic testing for the EWSR1-FLI-1 gene fusion. Also, we summarize the general CT performance of the EES.EES is also commonly accompanied by a lobulated contour and necrosis, which can lead to a heterogeneous enhancement pattern on CT examination.^[8–10]^ An imbalance associated with the tumor’s fast growth rate and insufficient blood supply could be why these tumors tend to exhibit severe necrosis. Second, almost all publications on EES report ill-defined borderlines^[10]^ and endoceliac lesions. Finally, EES often presents with a moderate degree of enhancement^[9]^ and absence of calcification.^[8]^ Although EES often misdiagnosed as intestinal mesenchymal tumor,^[11]^ an experienced doctor will notice that low-risk intestinal mesenchymal tumors are well-bordered masses and medium to high risk intestinal mesenchymal tumors present with a number of eccentric masses in the wall of the intestinal tract, which can be calcified. Pathology sections, immunohistochemistry and genetic testing are particularly important in the diagnosis of EES. However, the most common tumors with small round cell tumor (SRCT) morphology include Ewing sarcoma, synovial sarcoma, rhabdomyosarcoma (RMS), small-cell neuroendocrine carcinoma (SCNC), and desmoplastic SRCT, and the cytomorphologic distinction between these tumors is challenging. Therefore, we review several common SRCT morphologies of tumors, which includes the subtle morphologic differences among Ewing sarcoma, synovial sarcoma, RMS, SCNC, and DSRCT (Table 4). Additionally, each tumor subtype has its unique IHC profile and genetic alteration could be utilized for ancillary testing.

**Table 4:** Cytomorphological features of SRCT.

	Architecture	Helpful features	IHC	Gene-testing
Ewing sarcoma	Single cell, cell cluster	Possible neuroendocrine differentiation, possible nuclear molding	FLI-1, ERG, CD99	t(11;22) (q24;q12) (EWSR1-FLI-1) t(21;22) (q22;q12) (EWSR1-ERG)
Synovial sarcoma	Single cell, cell cluster	Nuclear folding, fusiform/tadpole forms	CK7, CK19, EMA, CD99, TLE-1	t(X;18) (p11.2;q11.2) SS18-SSX1, SS18-SSX2, rarely SS18-SSX4
RMS	Single dispersed cells	Spectrum of small to pleomorphic rhabdoid cells, multinucleated giant cells with “wreath-like” nuclear arrangements	Myogenin (Myf-4), MyoD1, desmin, SMA, myoglobin	t(2;13) (q35;q14) PAX3FOXO1 (Alveolar subtype)
SCNC	Linear chains of cells	Tight clusters of cells with molded nuclei around the periphery, nuclear molding, tumor cell cannibalism	Synaptophysin, chromogranin, CD56, epithelial markers (BerEP4, B72.3)	No well-defined characteristic mutation
DSRCT	Small cohesive clusters	Occasional molding, rare stromal tissue	CKs (CAM 5.2, AE1/AE3, EMA), WT-1, desmin, variable neural markers	t(11;22) (p13;q12) (EWSR1-WT-1)

ARMS = alveolar rhabdomyosarcoma, DSRCT = desmoplastic small round cell tumor, RMS = rhabdomyosarcoma, SCNC = small-cell neuroendocrine carcinoma, SRCT = small round cell tumors.

Only a few studies have examined the optimal treatment options and prognostic factors for EES.^[12]^ Although the optimal treatment and natural history of EES are still unknown,^[13–16]^ the treatment currently recommended by the National Comprehensive Cancer Network (NCCN) is localized treatment (surgery and/or radiotherapy) plus chemotherapy. According to Baci et al,^[17]^ neoadjuvant and adjuvant chemotherapy showed comparable results in patients with limited disease. Overall, chemotherapy improved overall survival and decreased the likelihood of postoperative recurrence.^[18]^ Current treatment regimens include alternating vincristine-adriamycin, cyclophosphamide, and isocyclophosphamide-etoposide every 3 weeks.^[19]^ Although chemotherapy regimens are necessary and have been shown to be effective, chemotherapy alone without surgery and/or radiation is not sufficient.^[20]^ EES is radiation sensitive; however, radiotherapy alone for local control has proven less effective over the years. Surgery may be performed when marginal or wide resection is possible.^[21]^ The ability to obtain adequate negative margins has the greatest impact on the local control of malignant tumors. When it is not possible to obtain wide margins due to the presence of fixed structures such as blood vessels and/or nerves, postoperative radiotherapy can be implemented to obtain better local control. However, according to the Cooperative European Ewing Sarcoma Study (CESS) and the European Intergroup Cooperative Ewing Sarcoma Study (EICESS) trials,^[22]^ intralesional resection plus radiotherapy did not yield superior local control rates compared to radiotherapy alone. In such cases, surgery may be avoided in favor of radiotherapy.^[22]^ Currently, definitive radiotherapy is only indicated for inoperable lesions, with a recommended dose of 54 to 55 Gy depending on the site of injury.^[23,24]^ However, larger tumors may require higher doses.^[23,24]^ Systemic chemotherapy was first considered for the patient described in this article. However, the patient was not treated with preoperative chemotherapy because the diagnosis of Ewing sarcoma was not confirmed on preoperative examination, which suggests that ultrasound-guided puncture biopsy can be performed to determine the nature of a mass of unknown origin.

Regarding prognosis, EES has a more favorable prognosis than the skeletal subtype, although factors affecting prognosis appear to be similar for both subtypes.^[25–27]^ Notably, the 5-year overall survival rate for localized EES is superior to that of localized skeletal Ewing sarcoma. Risk factors associated with a poorer prognosis for EES include older age,^[28]^ pelvic involvement,^[29]^ high WBC, elevated LDH, and low Hb at diagnosis.^[30,31]^ Initial tumor size is also a risk factor and is considered a strong prognostic factor for limited disease.^[28]^ However, in patients receiving neoadjuvant chemotherapy, histologic response is considered the strongest independent prognostic factor. Metastatic disease is a poor prognostic factor, with a 5-year overall survival rate of < 30% and < 20% for those with extrapulmonary involvement.^[32]^ Patients without metastases before treatment have a 5-year survival rate of up to 90% after chemotherapy. Of note, recurrent Ewing sarcoma, whether localized or metastatic, is almost always fatal.^[24]^ The patient in our report had high WBC, elevated LDH, low Hb, initial tumor size of approximately 13.2 × 9.9 × 15 cm, and multiple postoperative nodal metastatic, portending a poor prognosis.

In conclusion, we describe a case of intra-abdominal Ewing sarcoma that was misdiagnosed on abdominal CT, highlighting the need and difficulty of making a differential diagnosis in patients with such tumors. Histopathology is the key to the diagnosis of EES. Treatment of such patients is multimodal, meaning that treatment should begin with neoadjuvant chemotherapy, followed by local therapy, including surgery and radiation, and finally multiagent adjuvant chemotherapy. It is also important to determine the prognosis of the patient.

## Acknowledgments

The authors thank the patient and all the clinical staff who participated in the treatment of the patient.

## Author contributions

**Conceptualization:** Jianwu Wu, Xinwei Jiang.

**Methodology:** Cong Zhang, Song Li, Feng Wang.

**Writing – original draft:** Guang Yang.

**Writing – review & editing:** Lining Huang, Bo Huang, Jianwu Wu, Xinwei Jiang.

## References

[1] Van den Berg H Heinen RC Van der Pal HJ Merks JH. Extra-osseous Ewing sarcoma. Pediatr Hematol Oncol. 2009;26:175–85. 19437320 10.1080/08880010902855581

[2] Applebaum MA Worch J Matthay KK. Clinical features and outcomes in patients with extraskeletal Ewing sarcoma. Cancer. 2011;117:3027–32. 21692057 10.1002/cncr.25840 PMC3135782

[3] Chen Z Jiao Y Liu Z Yang J Sun J Wang P. Extraskeletal Ewing’s sarcoma: outcomes and CT features of endoceliac lesions. Transl Cancer Res. 2021;10:4065–75. 35116704 10.21037/tcr-21-607 PMC8798297

[4] Grier HE. The Ewing family of tumors. Ewing’s sarcoma and primitive neuroectodermal tumors. Pediatr Clin North Am. 1997;44:991–1004. 9286296 10.1016/s0031-3955(05)70541-1

[5] Aydinli B Ozturk G Yildirgan MI. Extraskeletal Ewing’s sarcoma in the abdominal wall: a case report. Acta Oncol. 2006;45:484–6. 16760186 10.1080/02841860500400987

[6] Downing JR Head DR Parham DM. Detection of the (11;22)(q24;q12) translocation of Ewing’s sarcoma and peripheral neuroectodermal tumor by reverse transcription polymerase chain reaction. Am J Pathol. 1993;143:1294–300. 8238248 PMC1887175

[7] Sorensen PH Lessnick SL Lopez-Terrada D. A second Ewing’s sarcoma translocation, t(21;22), fuses the EWS gene to another ETS-family transcription factor, ERG. Nat Genet. 1994;6:146–51. 8162068 10.1038/ng0294-146

[8] Somarouthu BS Shinagare AB Rosenthal MH. Multimodality imaging features, metastatic pattern and clinical outcome in adult extraskeletal Ewing sarcoma: experience in 26 patients. Br J Radiol. 2014;87:20140123. 24734938 10.1259/bjr.20140123 PMC4075565

[9] Gupta P Hari S Thulkar S. Imaging spectrum of peripheral primitive neuroectodermal tumours. Singapore Med J. 2013;54:463–2. 24005454 10.11622/smedj.2013155

[10] Li X Zhang W Song T Sun C Shen Y. Primitive neuroectodermal tumor arising in the abdominopelvic region: CT features and pathology characteristics. Abdom Imaging. 2011;36:590–5. 20959975 10.1007/s00261-010-9655-z

[11] Liao YS Chiang IH Gao HW. A mesenteric primary peripheral Ewing’s sarcoma/primitive neuroectodermal tumor with molecular cytogenetic analysis: report of a rare case and review of literature. Indian J Pathol Microbiol. 2018;61:248–51. 29676369 10.4103/IJPM.IJPM_546_17

[12] Ahmad R Mayol BR Davis M Rougraff BT. Extraskeletal Ewing’s sarcoma. Cancer. 1999;85:725–31. 10091746

[13] Biermann JS. Updates in the treatment of bone cancer. J Natl Compr Canc Netw. 2013;11(Suppl 5):681–3. 23704242 10.6004/jnccn.2013.0200

[14] Casali PG Bielack S Abecassis N. Bone sarcomas: ESMO-PaedCan-EURACAN clinical practice guidelines for diagnosis, treatment and follow-up. Ann Oncol. 2018;29(Suppl 4):iv79–95. 30285218 10.1093/annonc/mdy310

[15] Covelli HD Beekman JF Kingry RL. Extraskeletal Ewing’s sarcoma: prolonged survival with recurrence after operation. South Med J. 1980;73:1294–5. 7414394

[16] Rud NP Reiman HM Pritchard DJ Frassica FJ Smithson WA. Extraosseous Ewing’s sarcoma. A study of 42 cases. Cancer. 1989;64:1548–53. 2776115 10.1002/1097-0142(19891001)64:7<1548::aid-cncr2820640733>3.0.co;2-w

[17] Bacci G Balladelli A Forni C. Adjuvant and neoadjuvant chemotherapy for Ewing sarcoma family tumors in patients aged between 40 and 60: report of 35 cases and comparison of results with 586 younger patients treated with the same protocols in the same years. Cancer. 2007;109:780–6. 17219445 10.1002/cncr.22456

[18] Castex MP Rubie H Stevens MC. Extraosseous localized ewing tumors: improved outcome with anthracyclines: the French society of pediatric oncology and international society of pediatric oncology. J Clin Oncol. 2007;25:1176–82. 17401006 10.1200/JCO.2005.05.0559

[19] Womer RB West DC Krailo MD. Randomized controlled trial of interval-compressed chemotherapy for the treatment of localized Ewing sarcoma: a report from the Children’s Oncology Group. J Clin Oncol. 2012;30:4148–54. 23091096 10.1200/JCO.2011.41.5703 PMC3494838

[20] Donaldson SS. Ewing sarcoma: radiation dose and target volume. Pediatr Blood Cancer. 2004;42:471–6. 15049023 10.1002/pbc.10472

[21] Gaspar N Hawkins DS Dirksen U. Ewing sarcoma: current management and future approaches through collaboration. J Clin Oncol. 2015;33:3036–46. 26304893 10.1200/JCO.2014.59.5256

[22] Schuck A Ahrens S Paulussen M. Local therapy in localized Ewing tumors: results of 1058 patients treated in the CESS 81, CESS 86, and EICESS 92 trials. Int J Radiat Oncol Biol Phys. 2003;55:168–77. 12504050 10.1016/s0360-3016(02)03797-5

[23] Donaldson SS Torrey M Link MP. A multidisciplinary study investigating radiotherapy in Ewing’s sarcoma: end results of POG #8346. Pediatric Oncology Group. Int J Radiat Oncol Biol Phys. 1998;42:125–35. 9747829 10.1016/s0360-3016(98)00191-6

[24] Raney RB Asmar L Newton WA Jr. Ewing’s sarcoma of soft tissues in childhood: a report from the Intergroup Rhabdomyosarcoma Study, 1972 to 1991. J Clin Oncol. 1997;15:574–82. 9053479 10.1200/JCO.1997.15.2.574

[25] Lynch AD Gani F Meyer CF Morris CD Ahuja N Johnston FM. Extraskeletal versus skeletal Ewing sarcoma in the adult population: controversies in care. Surg Oncol. 2018;27:373–9. 30217290 10.1016/j.suronc.2018.05.016

[26] Cash T McIlvaine E Krailo MD. Comparison of clinical features and outcomes in patients with extraskeletal versus skeletal localized Ewing sarcoma: a report from the Children’s Oncology Group. Pediatr Blood Cancer. 2016;63:1771–9. 27297500 10.1002/pbc.26096 PMC4995129

[27] Takenaka S Naka N Obata H. Treatment outcomes of Japanese patients with Ewing sarcoma: differences between skeletal and extraskeletal Ewing sarcoma. Jpn J Clin Oncol. 2016;46:522–8. 27008849 10.1093/jjco/hyw032

[28] Brinkhuis M Wijnaendts LC van der Linden JC. Peripheral primitive neuro-ectodermal tumour and extra-osseous Ewing’s sarcoma; ahistological, immunohistochemical and DNA flow cytometricstudy. Virchows Arch. 1995;425:611–6. 7697218 10.1007/BF00199351

[29] Lee JA Kim DH Lim JS. Soft-tissue Ewing sarcoma in a low-incidence population: comparison to skeletal Ewing sarcoma for clinical characteristics and treatment outcome. Jpn J Clin Oncol. 2010;40:1060–7. 20513751 10.1093/jjco/hyq080

[30] Orr WS Denbo JW Billups CA. Analysis of prognostic factors in extraosseous Ewing sarcoma family of tumors: review of St. Jude Children’s Research Hospital experience. Ann Surg Oncol. 2012;19:3816–22. 22739653 10.1245/s10434-012-2458-4

[31] Biswas B Shukla NK Deo SV. Evaluation of outcome and prognostic factors in extraosseous Ewing sarcoma. Pediatr Blood Cancer. 2014;61:1925–31. 25132242 10.1002/pbc.25095

[32] Haeusler J Ranft A Boelling T. The value of local treatment in patients with primary, disseminated, multifocal Ewing sarcoma (PDMES). Cancer. 2010;116:443–50. 19924786 10.1002/cncr.24740

